# Marker-Assisted Introgression and Stacking of Major QTLs Controlling Grain Number (*Gn1a*) and Number of Primary Branching (*WFP*) to NERICA Cultivars

**DOI:** 10.3390/plants10050844

**Published:** 2021-04-22

**Authors:** Vincent P. Reyes, Rosalyn B. Angeles-Shim, Merlyn S. Mendioro, Ma. Carmina C. Manuel, Ruby S. Lapis, Junghyun Shim, Hidehiko Sunohara, Shunsaku Nishiuchi, Mayumi Kikuta, Daigo Makihara, Kshirod K. Jena, Motoyuki Ashikari, Kazuyuki Doi

**Affiliations:** 1 Graduate School of Bioagricultural Sciences, Nagoya University, Nagoya 464-8601, Japan; reyes.vincent.pamugas@f.mbox.nagoya-u.ac.jp (V.P.R.); taichung65@yahoo.co.jp (H.S.); s_nishi@agr.nagoya-u.ac.jp (S.N.); 2 Novel Gene Resources Laboratory, Plant Breeding Division, International Rice Research Institute, DAPO Box 7777, Metro Manila, Philippines; Rosalyn.shim@ttu.edu (R.B.A.-S.); r.lapis@irri.org (R.S.L.); kshirodjena86@gmail.com (K.K.J.); 3 Graduate School, University of the Philippines Los Banos, College, Laguna 4031, Philippines; msmendioro@up.edu.ph (M.S.M.); mcmanuel1@up.edu.ph (M.C.C.M.); 4 Current address: Department of Plant and Soil Science, College of Agricultural Sciences and Natural Resources, Texas Tech University, Lubbock, TX 79409, USA; Junghyun.shim@ttu.edu; 5 Bioscience and Biotechnology Center, Nagoya University, Nagoya 464-8601, Japan; ashi@agr.nagoya-u.ac.jp; 6 Applied Social System Institute of Asia, Nagoya University, Nagoya 464-8601, Japan; mkikuta@agr.nagoya-u.ac.jp; 7 International Center for Research and Education in Agriculture, Nagoya University, Nagoya 464-8601, Japan; makihara@agr.nagoya-u.ac.jp

**Keywords:** *Gn1a*, *WFP*, marker-assisted backcrossing, genotyping-by-sequencing, crop improvement, molecular breeding, NERICA, rice yield

## Abstract

The era of the green revolution has significantly improved rice yield productivity. However, with the growing population and decreasing arable land, rice scientists must find new ways to improve rice productivity. Although hundreds of rice yield-related QTLs were already mapped and some of them were cloned, only a few were utilized for actual systematic introgression breeding programs. In this study, the major yield QTLs *Grain Number 1a* (*Gn1a*) and *Wealthy Farmer’s Panicle* (*WFP*) were introgressed and stacked in selected NERICA cultivars by marker-assisted backcross breeding (MABB). The DNA markers RM3360, RM3452, and RM5493 were used for foreground selection. At BC_3_F_4_ and BC_3_F_5_ generation, a combination of marker-assisted selection and phenotypic evaluation were carried out to select lines with target alleles and traits. Further, genotyping-by-sequencing (GBS) was conducted to validate the introgression and determine the recurrent parent genome recovery (RPGR) of the selected lines. The *Gn1a* and/or *WFP* introgression lines showed significantly higher numbers of spikelets per panicle and primary branching compared to the recurrent parents. In addition, lines with *Gn1a* and/or *WFP* alleles were comparatively similar to the recurrent parents (RP) in most yield-related traits. This study demonstrates the success of utilizing yield QTLs and marker-assisted selection to develop and improve rice cultivars.

## 1. Introduction

Rice (*Oryza sativa* L.) is the main staple for almost half of the world’s population. Although mostly consumed in Asia, it is also considered the fastest-growing staple in Africa and Latin America. In sub-Saharan Africa (SSA), rice consumption has significantly increased over the years. This is due to factors such as economic growth, population growth, and changing consumer preference. In SSA countries such as Kenya, Nigeria, and Tanzania, people are changing their food preferences from tubers to rice as their income rises [1]. Given the tight supply–demand in rice across the globe and the decline in rice lands in Asia, being dependent on rice import is seen to be a significant threat to SSA’s food security. Therefore, it is necessary to develop and improve their existing rice varieties [2].

Most agronomic traits such as yield and yield-related components are polygenic and highly influenced by the environment. In rice, panicle is one of the crucial traits for improving rice grain yield. The vital components of rice panicle are the number of spikelets per panicle (NSPP), panicle length (PL), number of primary branches per panicle (PBPP), and number of secondary branches (SBPP) [3]. To understand the mechanism and nature of these agronomic traits, genetic and molecular dissection is needed [4].

The advent of genome sequencing has significantly influenced rice breeding studies. The completion of rice genome sequencing has led to the identification, isolation, and characterization of agronomically important traits and their application in varietal improvement [5,6]. To date, several quantitative trait loci (QTL) have been demonstrated to influence rice yield and yield-related traits such as heading date (*Hd1*, *Ghd7*, and *Ehd1* [7,8,9]), grain size and weight (*GS3*, *GW2*, and *GW5* [10,11,12]), grain number (*Gn1a* and *DEP1* [13,14]), and panicle branching (*WFP*/*IPA1* [15]). The major QTL *grain number 1a* (*Gn1a*) was first identified in the high-yielding rice variety Habataki. It encodes cytokinin oxidase/dehydrogenase (*OsCKX2*), an enzyme that degrades bioactive cytokinin. When the expression of *OsCKX2* is reduced, cytokinin accumulates, resulting in an increase in the branching of inflorescence [13]. On the other hand, *Wealthy Farmer’s Panicle* (*WFP*) was first isolated from the rice line ST12. This gene encodes *SQUAMOSA promoter binding protein-like 14* (*OsSPL14*), which is then regulated by microRNA *OsmiR156.* An increase in *OsSPL14* expression during the vegetative stage suppresses tillering and enhances panicle branching. In rice line ST12, an abundance of *OsSPL14* transcripts is regulated by a heritable epigenetic mechanism [15]. The major QTLs *Gn1a* and *WFP* were previously used in some breeding programs for improvement of the *indica* and *japonica* rice cultivars [16,17,18]. However, the effects of introgression and stacking of these QTLs in an interspecific background, such as NERICA, have yet to be evaluated.

The concept of backcrossing is to transfer the specific allele to the target locus. However, traditional backcrossing is laborious and requires more generations to obtain lines with high RPGR. The inclusion of DNA markers in the backcrossing program is called MABB. As compared to traditional backcrossing, MABB makes use of DNA markers that are tightly linked or flank the target locus [19]. This shifts the selection from phenotype to genotype which is far more efficient and accurate for selecting the target trait [20]. In addition, MABB greatly accelerates the identification and selection of lines with high RPGR, thereby reducing the number of required backcrossing. This method has been proven successful in some breeding programs for biotic stresses [21,22] and abiotic stresses [23].

Over the years, the use of DNA markers in breeding programs has transitioned from the traditional simple sequence repeat (SSR) to single nucleotide polymorphic (SNP) markers. The shift in marker preference is due to the advances in the field of sequencing technology [24,25]. GBS is a reduced representation library method which allows highly multiplexed sequencing of DNA samples. Compared to other genotyping approaches, GBS gives a greater number of markers and more depths per read which is advantageous for detecting heterozygous regions [26]. This method has been successfully used in plant breeding applications such as genetic resource development, QTL mapping, and background genome selection [27,28]. However, utilizing this method for yield improvement has remained marginal.

Introducing beneficial genes alone cannot efficiently improve the target traits due to limiting factors such as genetic and environmental effects [29]. To overcome these limiting factors, critical evaluation of the effects of the target allele in different genomic backgrounds is essential in breeding programs. Therefore, the main objectives of this study are the following: (i) improve the grain number of widely preferred NERICA and related cultivars by introgression of the major QTLs, *Gn1a,* and *WFP,* (ii) identify promising lines with improved grain number and primary branching, and (iii) evaluate the effect on the yield and yield-related components of rice. The data obtained in this study is part of the Wonder Rice Initiative for Food Security and Health (WISH) project [30].

## 2. Results

### 2.1. Marker-Assisted Backcrossing for Development of WISH Lines

**Figure 1:**
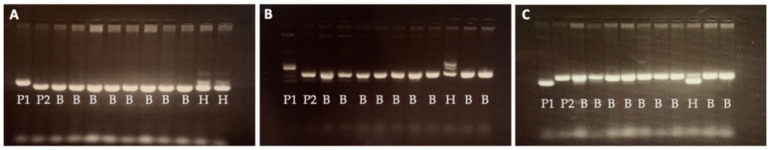
Marker-assisted selection of progeny (BC_3_F_4_) using RM markers tightly linked to *Gn1a* and flanking the *WFP*. (**A**) Progeny were screened using RM3360 for the *Gn1a* allele; (**B**) progeny were screened using RM5493; (**C**) progeny were screened using RM3452 for the *WFP* allele. A, B, and H refer to the recipient (P1), donor (P2), and heterozygous genotypes, respectively.

The initial crosses of the materials were developed at Nagoya University, Japan. MABB was employed to transfer the major QTLs *Gn1a* and/or *WFP* from the donor lines ST12 [15] and ST6 [31] to NERICA and WAB cultivars. The RM3360, which is tightly linked to *Gn1a*, and RM3452 and RM5493, which flank the *WFP* locus [32], were used for foreground selection. Progeny at BC_1_F_1_ generation were shipped to the International Rice Research Institute (IRRI) for further backcrossing and generation advancement. The selected BC_1_F_1_ plants were advanced to BC_3_F_1_ and subsequently followed through selfing generations to identify plants carrying homozygous alleles at the target locus. The first two lanes on the gel images (Figure 1) represent the recurrent and donor parent genotypes, respectively. At BC_3_F_4_, using the RM3360 *(*Figure 1A), RM 5493 (Figure 1B), and RM 3452 (Figure 1C), lines with homozygous genotypes were evaluated for target traits and further genotyped using GBS.

### 2.2. Recurrent Parent Genome Recovery Analysis on Selected Improved Lines

To validate the introgression and analyze the RPGR, the selected 192 candidate lines were genotyped through GBS following the method by Poland et al. (2012) [33], with a combination of restriction enzyme of *Kpn*I and *Msp*I. A total of 141.8 Gb sequence data were generated from the 192 lines. After the initial filtering and processing, a total of 55,082 SNPs were obtained. Another set of filtering was executed which trimmed down the SNPs to an average of 2500 per genetic background. Finally, after error correction and manual curation, an average of 1400 high-quality SNPs were retained and used for further analysis. The total number of SNPs and range of RPGR in each genetic background are summarized in Table 1.

In the genetic background of NERICA1, lines carrying the *Gn1a-ST12*, *WFP-ST12*, and a combination of both alleles had an RPGR ranging from 85.4–98%. The WISH line 1:1-3-4-3-3 (NERICA1 + Gn1a-ST12-1) carrying the *Gn1a-ST12* allele had the highest RPGR at 98%. In lines carrying the *WFP-ST12*, WISH 1:4-15-1-7-1 (NERICA1+WFP-ST12-2) had the highest RPGR at 97.3%, and for lines carrying both alleles, WISH 1:6-12-8-12-4 (NERICA1 + Gn1a + WFP-ST12-1) had the highest RPGR at 92.2%. In the genetic background of NERICA6, WISH 14:23-7-6-4-7 (NERICA6 + Gn1a-ST12-1) had an RPGR of 94.8%. The lines in the genetic background of WAB56-104 with a single QTL introgression and stacked QTLs had an RPGR ranging from 77–96.9%. In lines carrying the *Gn1a-ST12* allele, WISH 22:1-4-1-6-7 (WAB56-50 + Gn1a-ST12-3) had the highest RPGR at 96.4%, while in lines carrying the *WFP-ST12* allele, WISH 22:5-3-3-1-2 (WAB56-104 + WFP-ST12-4) had the highest RPGR at 96.9%. On the other hand, in lines carrying both alleles, WISH 22:5-6-1-3-7 (WAB56-104 + Gn1a + WFP-ST12-2) showed the highest RPGR at 95.1%. In the genetic background of WAB56-50, lines were identified with an RPGR ranging from 88.3–96.7%. The WISH line 23:1-7-4-16-1-4 (WAB56-50 + Gn1a-ST12-2), carrying the *Gn1a-ST12* allele, showed the highest RPGR at 96.7%. In lines carrying the *WFP-ST12* allele, WISH 23:1-4-4-16-2-3 had the highest RPGR at 96%. Lastly, WISH 23:1-12-10-20-1-5 (WAB56-50 + Gn1a + WFP-ST12-1) showed the highest RPGR at 94.4%

**Figure 2:**
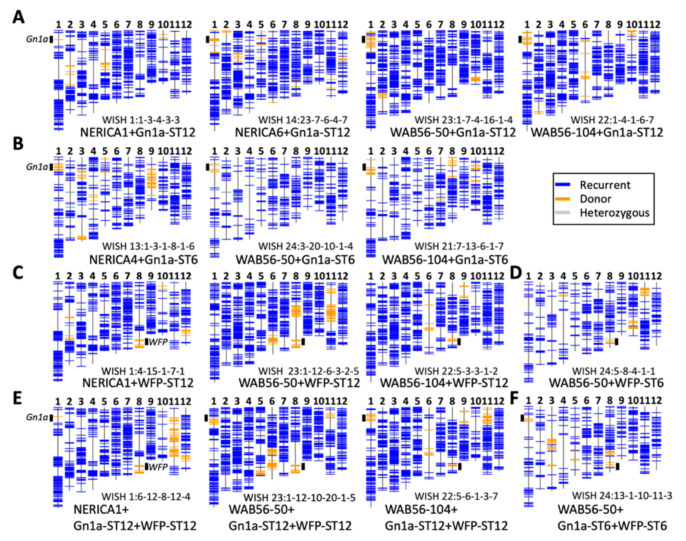
Marker genotypes of the representative lines. Genotypes of GBS markers are represented with horizontal color lines. Chromosomes and marker positions are drawn to scale of physical lengths, and positions of *Gn1a* and *WFP* are shown in short bold black lines. (**A**) Selected lines with *Gn1a-ST12*, (**B**) selected lines with *Gn1a-ST6*, (**C**) selected lines with *WFP-ST12*, (**D**) selected lines with *WFP-ST6*, (**E**) selected lines with *Gn1a-ST12* + *WFP-ST12*, (**F**) selected line with *Gn1a-ST6* + *WFP-ST6*.

RPGR was also evaluated in lines carrying an introgression from the donor parent ST6. The lines in the genetic background of NERICA4 x ST6 had an RPGR ranging from 84.5–87.7% with WISH 13:1-3-1-8-1-6 (NERICA4+Gn1a-ST6-1) having the highest RPGR at 87.7%. In the genetic background of WAB56-104, WISH 21:7-13-5-1-5 (WAB56-50 + Gn1a-ST6-4) and WISH 21:7-13-6-1-7 (WAB56-104 + Gn1a-ST6-3) showed the highest RPGR at 93%. The WISH lines 24:3-14-4-1-3 (WAB56-50 + Gn1a-ST6-1) and 24:3-20-10-1-4 (WAB56-50 + Gn1a-ST6-2) showed the highest RPGR at 95%. In the same genetic background, WISH 24:5-8-4-1-1 (WAB56-50 + WFP-ST6-2) showed an RPGR of 94%. In lines carrying both alleles, the WISH line 24:13-1-10-11-3 (WAB56-50 + Gn1a + WFP-ST6-1) showed the highest RPGR at 92.1%. The graphical genotypes of lines with the highest RPGR in each genetic background are presented in Figure 2.

### 2.3. Effects of Gn1a Allele on Grain Number

**Figure 3:**
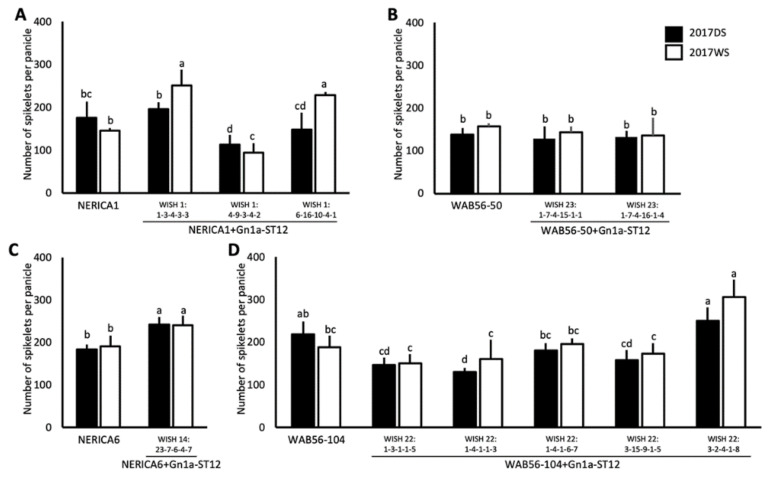
Number of spikelets per panicle (mean ± SD) of *Gn1a-ST12* introgression lines in the genetic backgrounds of NERICA1 (**A**), WAB56-50 (**B**), NERICA6 (**C**), and WAB56-104 (**D**). Solid and white bars show the results in 2017DS and 2017WS, respectively. Different letters above the bar graphs indicate significant difference at *p* < 0.05.

The effects of *Gn1a* on yield and yield-related components were evaluated during the dry season (DS) and wet season (WS) of 2017. In the genetic background of NERICA1, two out of the three lines were observed to have a significant improvement in NSPP during the WS of 2017. The WISH line 1:1-1-3-4-3-3 (NERICA1 + Gn1a-ST12-1) significantly improved by 72.3%, while the WISH line 1:6-16-10-4-1 (NERICA1 + Gn1a-ST12-3) significantly improved by 56.74% as compared to that of NERICA1 (Figure 3A). In the genetic background of NERICA6, the NSPP of WISH line 14:23-7-6-4-7 (NERICA6 + Gn1a-ST12-1) significantly improved by 32.4% during DS and 25.9% during WS (Figure 2B). No significant improvement was observed in the genetic background of WAB56-50 × ST12 (Figure 2C). Among the five introgression lines in the genetic background of WAB56-104 × ST12, only the WISH line 22:3-2-4-1-8 (WAB56-50 + Gn1a-ST12-5) showed a significant improvement in NSPP by 14.2% during DS and 62.5% during WS of 2017 (Figure 3C).

**Figure 4:**
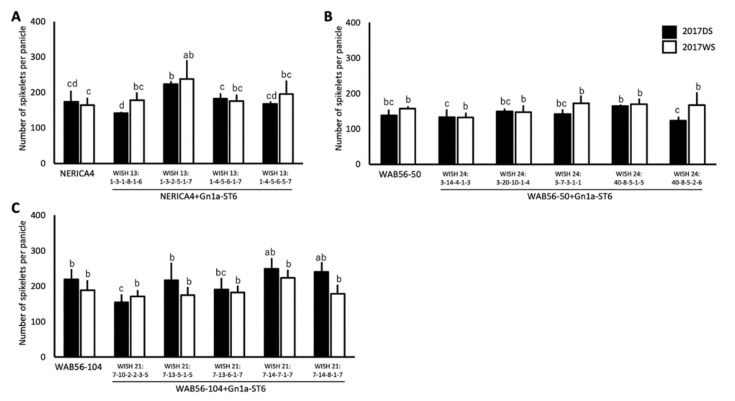
Number of spikelets per panicle (mean ± SD) of *Gn1a-ST6* introgression lines in the genetic backgrounds of NERICA4 (**A**), WAB56-50 (**B**), and WAB56-104 (**C**). Solid and white bars show the results in 2017DS and 2017WS, respectively. Different letters above the bar graphs indicate significant difference at *p* < 0.05.

Evaluation of *Gn1a-ST6* in the genetic backgrounds of NERICA4, WAB56-50, and WAB56-104 was also carried out. In the genetic background of NERICA4, two lines were observed to have a significantly improved NSPP. The WISH line 13:1-3-2-5-1-7 (NERICA4 + Gn1a-ST6-2) was observed to have a significantly improved NSPP by 28.10% during DS, and 45.13% during the WS of 2017. On the other hand, the NSPP of the WISH line 13:4-5-6-1-7 (NERICA4 + Gn1a-ST6-3) significantly improved by 4.9% during DS (Figure 4A). The same occurred with WAB56-50 × ST12; no significant improvement was observed in the *Gn1a* introgression lines of WAB56-50 x ST6 (Figure 4B). A consistent improvement in NSPP was observed in the WISH line 21:7-14-7-1-7 (WAB56-104 + Gn1a-ST6-4). The NSPP of this WISH line significantly improved by 14.08% during DS and 18.69% during WS (Figure 4C).

### 2.4. Effects of WFP Allele on Primary Branching Number and Grain Number

**Figure 5:**
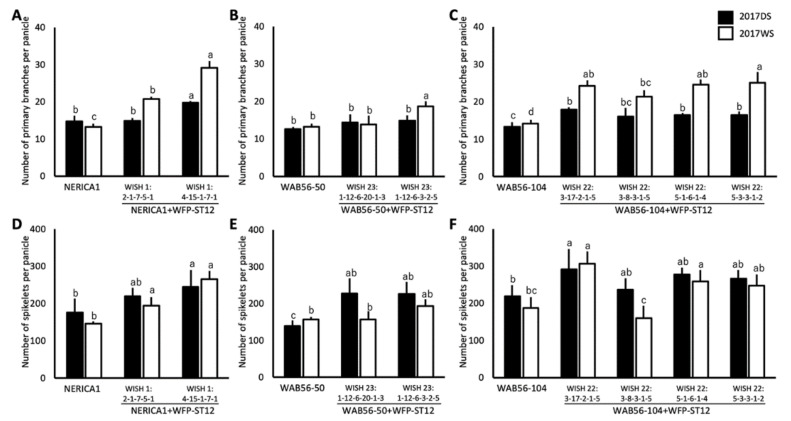
Number of primary branches per panicle (**A**–**C**) and numbers of spikelets per panicle (**D**–**F**) (mean ± SD) of *WFP*-*ST12* introgression lines in the genetic backgrounds of NERICA1 (**A** and **D**), WAB56-50 (**B** and **E**), and WAB56-104 (**C** and **F**). Solid and white bars show the results in 2017DS and 2017WS, respectively. Different letters above the bar graphs indicate significant difference at *p* < 0.05.

To determine the effects of *WFP* allele, PBPP and NSPP of *WFP-ST12* and *WFP-ST6* introgression lines were evaluated. In the genetic background of NERICA1 × ST12, two lines with a significant improvement on PBPP were identified. The WISH line 1:4-15-1-7-1 (NERICA1 + WFP-ST12-2) was observed to have a 33.78% increase in PBPP during DS and 118% during WS, while the WISH line 1:2-1-7-5-1 (NERICA1 + WFP-ST12-1) was observed to have a significant improvement in PBPP by 55.51% during WS (Figure 5A). Out of the two lines, only the WISH line 1:4-15-1-7-1 showed a consistent improvement on NSPP, by 39.05% during DS and 82.25% during WS (Figure 5D). In WAB56-104 × ST12, the WISH lines 22:3-17-2-1-5 (WAB56-104 + WFP-ST12-1), WISH 22:3-8-3-1-5 (WAB56-104 + WFP-ST12-2), WISH 22:5-1-6-1-4 (WAB56-104+WFP-ST12-3), and WISH 22:5-3-3-1-2 (WAB56-104 + WFP-ST12-4) were observed to have a significantly improved PBPP (Figure 5C). The WISH line 22:3-17-2-1-5 (WAB56-104 + WFP-ST12-1) was observed to have improvement in PBPP by 34.89% during DS and 73.17% during WS. This line was also observed to have a higher NSPP compared to the RP, by 33.77% during DS and 62.70% during WS 2017 (Figure 5F). In WAB56-50 × ST12 genetic background, only the WISH line 23:1-12-6-3-2-5 (WAB56-50 + WFP-ST12-2) showed a significant improvement in PBPP, by 40.69% during WS (Figure 5B). In terms of NSPP, the WISH line 23:1-12-6-20-1-3 (WAB56-50 + WFP-ST12-1) showed a significant improvement by 63.35% and the WISH line 23-1-12-6-3-2-5 (WAB56-50 + WFP-ST12-2) by 62.62% during DS (Figure 5E). The graphical image of the primary branches per panicle and spikelets per panicle of the *WFP*-*ST12* introgression lines and their respective RPs are summarized in Figure 5.

**Figure 6:**
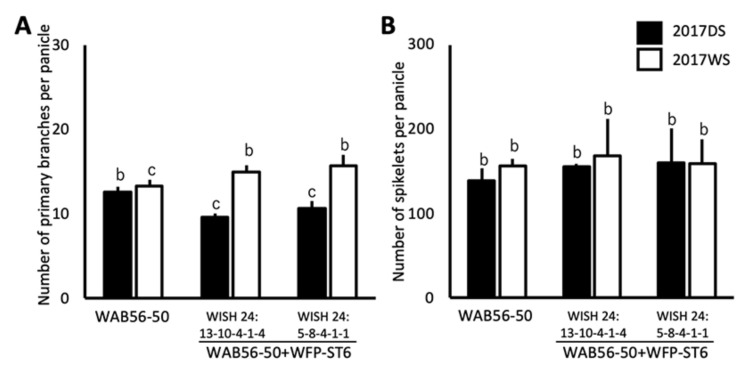
Number of primary branches per panicle (**A**) and numbers of spikelets per panicle (**B**) (mean ± SD) of *WFP-ST6* introgression lines in the genetic background of WAB56-50. Solid and white bars show the results in 2017DS and 2017WS, respectively. Different letters above the bar graphs indicate significant difference at *p* < 0.05.

The *WFP-ST6* introgression lines were evaluated for the same traits. A significant improvement was observed in PBPP of WISH 24:13-10-4-1-4 (WAB56-50 + WFP-ST6-1) and WISH 24:5-8-4-1-1 (WAB56-50 + WFP-ST6-2) during WS (Figure 6A). However, no significant improvement was observed in the NSPP of both lines (Figure 6B).

### 2.5. Effects of Stacking of Gn1a + WFP on Spikelet Number and Primary Branching

**Figure 7:**
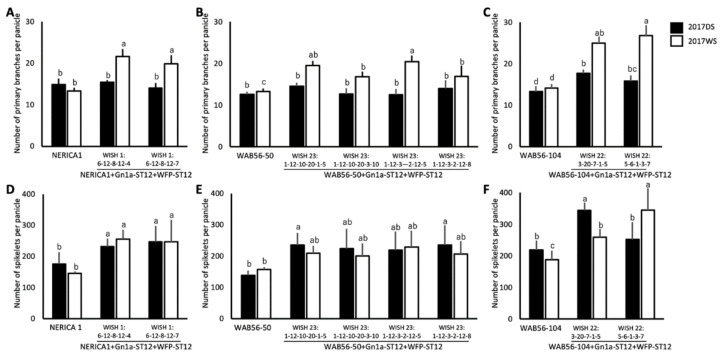
Number of primary panicle branches (**A**–**C**) and number of spikelets per panicle (**D**–**F**) (mean ± SD) of stacked introgression lines of *Gn1a-ST12* and *WFP*-*ST12* in the genetic backgrounds of NERICA1 (**A** and **D**), WAB56-50 (**B** and **E**), and WAB56-104 (**C** and **F**). Solid and white bars show the results in 2017DS and 2017WS, respectively. Different letters above the bar graphs indicate significant difference at *p* < 0.05.

In this study, the yield QTLs *Gn1a* and *WFP* were stacked in NERICA1, WAB56-50, and WAB56-104. In the genetic background of NERICA1 × ST12, the WISH lines 1:6-12-8-12-4 (NERICA1 + Gn1a+WFP-ST12-1) and 1:6-12-8-12-7 (NERICA1 + Gn1a + WFP-ST12-2) showed a significant improvement in PBPP by 62.57% and 49.51%, respectively (Figure 7A). Consistently, the WISH line 1:6-12-8-12-4 (NERICA1+Gn1a + WFP-ST12-1) showed a significant improvement in NSPP by 31.88% during DS and 74.99% during WS (Figure 7D), while the WISH line 1:6-12-8-12-7 (NERICA1 + Gn1a + WFP-ST12-2) significantly improved by 39.99% during DS and 69.14% during the WS (Figure 7D). For WISH lines in the genetic background of WAB56-50 × ST12, WISH lines 23:1-12-10-20-1-5 (WAB56-50 + Gn1a + WFP-ST12-1), WISH 23-1-12-10-20-3-10 (WAB56-50 + Gn1a + WFP-ST12-2), WISH 23:1-12-3-2-12-5 (WAB56-50 + Gn1a + WFP-ST12-3), and WISH 23:1-12-3-2-12-8 (WAB56-50 + Gn1a + WFP-ST12-4) showed a significant improvement in PBPP during WS (Figure 7B). However, out of these four lines, only the WISH lines 23:1-12-10-20-1-5 (WAB56-50 + Gn1a + WFP-ST12-1) and 23:1-12-3-2-12-8 (WAB56-50 + Gn1a + WFP-ST12-4) showed a significant increase in NSPP by 70% during DS (Figure 7E). In the genetic background of WAB56-104 × ST12, the WISH lines 22:3-20-7-1-5 (WAB56-104 + Gn1a + WFP-ST12-1) and WISH 22:5-6-1-3-7 (WAB56-104 + Gn1a + WFP-ST12-2) showed a significant improvement in PBPP and NSPP. The WISH line 22:5-6-1-3-7 (WAB56-104 + Gn1a + WFP-ST12-2) showed a significant improvement in PBPP by 19.29% during DS and 89.17% during the WS of 2017 (Figure 7C), while the WISH line 22:3-20-7-1-5 (WAB56-104 + Gn1a + WFP-ST12-1) showed a significant improvement in PBPP by 33.16% during DS and 76.93% during WS of 2017 (Figure 7C). In terms of NSPP, the WISH line 22:5-6-1-3-7 (WAB56-104 + Gn1a + WFP-ST12-2) showed a significant improvement by 83.88% only during DS (Figure 7F). The NSPP of the WISH line 22:3-20-7-1-5 (WAB56-104 + Gn1a + WFP-ST12-1) significantly improved by 56.79% during DS and 37.76% during WS (Figure 7F).

**Figure 8:**
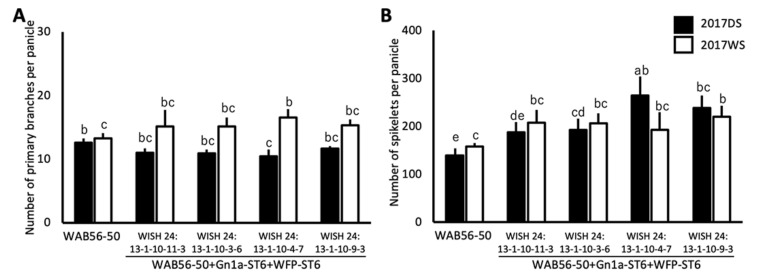
Number of primary panicle branches (**A**) and number of spikelets per panicle (**B**) (mean ± SD) of stacked introgression lines of *Gn1a*-*ST6* and *WFP*-*ST6* in the genetic background of WAB56-50. Solid and white bars show the results in 2017DS and 2017WS, respectively. Different letters above the bar graphs indicate significant difference at *p* < 0.05.

In the WAB56-50 × ST6 genetic background, no significant improvement was observed in PBPP (Figure 8A). However, three lines showed a significant improvement in NSPP. The WISH lines 24:13-1-10-3-6 (WAB56-50 + Gn1a + WFP-ST6-2) and 24:13-1-10-4-7 (WAB56-50 + Gn1a + WFP-ST6-3) showed a significant improvement in NSPP by 38.96% and 90.85%, respectively, during DS (Figure 8B). On the other hand, the WISH line 24:13-1-10-9-3 (WAB56-50 + Gn1a + WFP-ST6-4) showed a constant improvement in NSPP by 71.71% during DS and 39.68% during WS (Figure 8B).

### 2.6. Agronomic Performance of WISH Introgression Lines

In the current study, seven other major agronomic traits were evaluated for two seasons: days to heading (DTH), plant height (PH), tiller number per plant (TNPP), PL, SBPP, percent fertility (PF), and thousand-grain weight (TGW).

The WISH lines with *Gn1a-ST12* in the genetic background of NERICA1 and WAB56-104 showed a significant improvement in SBPP by 32.2–105% during DS and 72.7–117.2% during WS (Supplementary Table S1), while the WISH lines carrying the *Gn1a-ST6* allele in the genetic backgrounds of NERICA4, WAB56-104, and WAB56-50 showed a significant improvement in SBPP by 32.5–101.2% during DS and 27.85–54.4% during WS (Supplementary Table S2).

The performance of other agronomic traits observed in this study was comparatively similar to that of the respective recurrent parents. However, some lines evaluated were significantly different. The significant difference observed across these phenotypes varies among the sister lines and across seasons. For example, in the genetic background of NERICA1, the WISH line 1:4-9-3-4-2 (NERICA1 + Gn1a-ST12-2) was significantly shorter as compared to its RP, but only during WS. The WISH 23:1-12-6-3-2-5 (WAB56-50 + WFP-ST12-2) was observed to be heavier, as compared to the RP, but only during WS (Supplementary Table S3). The same was observed in WISH lines with stacked yield QTLs. The WISH line 23:1-12-10-20-3-10 (WAB56-50 + Gn1a-ST12 + WFP-ST12-2) was observed to have significantly higher PF, but only during DS (Supplementary Table S5).

## 3. Discussion

Marker-assisted breeding has served as a hallmark of an efficient way to improve several traits across different crops. To improve the existing rice cultivars in Africa, the WISH project made use of an MABB scheme to transfer and stack the yield QTLs *Gn1a* and *WFP*. In this study, NERICA lines and their WAB progenitors were used as the recipient parents of yield QTLs *Gn1a* and *WFP*. The NERICA plant materials have big and heavy panicles but with a fewer number of tillers [34]. These lines were also reported to have tolerance in some biotic and abiotic stresses. On the other hand, the WAB progenitors were reported to have desirable qualities such as drought tolerance, weed competitiveness, and disease tolerance [34].

The effects of the introgression of *Gn1a* alleles in this study coincide with the findings of Sakamoto et al. (2008) [35]. Their findings showed that a near-isogenic line (NIL) carrying *Gn1a* had the same number of PBPP but developed a higher number of SBPP on both primary branches and at the panicle base. The developed WISH lines in this study with *Gn1a-ST12* and *Gn1a-ST6* introgression showed a significant increase in SBPP by as much as 13–117% in the genetic backgrounds of NERICA1, NERICA4, NERICA6, and WAB56-104. The improvement in SBPP in the WISH lines significantly increased the NSPP by as much as 4.9–72.3% in the same genetic backgrounds. In a previous study of Ashikari et al. (2005) [13], the major QTLs *Gn1a* + *Gn1b* from Habataki were able to increase grain productivity by as much as ~45% in the Koshihikari background. In 2017, Feng et al. [18] showed that introgression of *Gn1a* allele from the donor parent GKBR improved the yield per plant of a *japonica* rice cultivar Kongyu 131 by 8.3% and 11.9% in two locations in Heilongjian, China. The effects of the *Gn1a* allele on NERICA and WAB56-104 introgression lines that we developed are the same as the results of other *Gn1a* introgression studies. In a previous study of Kim et al. (2018) [16], introgression of *Gn1a* alleles from Habataki, ST12, and ST6 were found to be ineffective in some *indica* rice cultivars as they have the same type of *Gn1a* allele with the donor parents. The same may hold true in the WISH lines in the genetic background of WAB56-50 × ST12.

The introgression of the *WFP* allele showed a significant improvement in PBPP in the genetic backgrounds of NERICA1, WAB56-104, and WAB56-50 by 33.77–118%. This significant increase in PBPP resulted in an increase in NSPP by 33.77–82.25%. However, the lines carrying the *WFP-ST12* and *WFP-ST6* alleles showed a significant reduction in TNPP (Supplementary Tables S3 and S4). The results obtained in this study coincide with the findings of previous studies on *WFP*. In the study of Jiao et al. (2010) [36], *OsSPL14* from the rice line Shaoniejing, a *japonica* cultivar, significantly increased the PBPP and NSPP of Taichung 1, an *indica* rice variety. However, TNPP was greatly reduced. Miura et al. (2010) [15], on the other hand, used the *OsSPL14* allele of the rice line ST12 and Aikawa. Their result showed that lines carrying the *WFP-ST12* allele significantly improved PBPP and NSPP without a decrease in TNPP. However, the lines carrying the *WFP-Aikawa* allele showed significant improvement in PBPP and NSPP, but with significant reduction in TNPP [15]. In the recent study by Kim et al. (2018) [16], *WFP*-*ST12* and *WFP-Aikawa* were introgressed to an *indica* rice cultivar. Their result showed that *WFP-ST12* significantly improved PBPP and NSPP without reducing the TNPP. In the study of Yamada et al. (2020) [17], introgression of the *WFP-ST12* allele in the genetic background of IRBB60 significantly improved the PBPP. The developed WISH lines in our study deviate from the findings of Miura et al. (2010) [15] and Kim et al. (2018) [16], wherein TNPP in WISH lines with *WFP-ST12* and *WFP-ST6* were significantly reduced. The reduction in TNPP was previously associated with *DEP1* by Lu et al. (2013) [37]. It was characterized that trade-off pleiotropy between PBPP and TNPP happens between *IPA1*/*WFP*/*OsSPL14* and *DEP1*. The *IPA1* functions as a positive regulator to *DEP1,* increasing PH and PL. In addition, *IPA1/OsSPL14* can also interact and directly bind to several genes that regulate plant architecture such as *OsTB1*, *PIN1b*, *SLR*, *LOG*, and *LAX* [37,38].

Gene stacking has been applied mostly for biotic and abiotic stresses. However, only a few breeding programs apply gene stacking for yield traits. In this study, stacking of the yield QTLs from the donor parents ST12 and ST6 showed a significant improvement in PBPP, SBPP, and NSPP. However, the improvement observed in PBPP, SBPP, and NSPP was on par with those in lines with single QTL introgression. This result coincides with the findings of Kim et al. (2018) [16], wherein the yield productivity of stacked lines was not significantly different from that of the lines carrying a single gene introgression. In addition, the WISH lines with stacked QTLs had a significantly lower number of TNPP (Supplementary Tables S5 and S6). The drastic reduction in the TNPP could be attributed to the *WFP* allele.

The background genome recovery in this study was determined by genotyping-by-sequencing. As compared to SSR markers, GBS has a greater number of markers per sample and a greater number of alleles per marker [39]. In addition, GBS offers a cheaper cost per data point compared to other traditional DNA markers [40]. The number of SNPs obtained for RPGR analysis showed variation per cross combination. For example, in the cross combination of NERICA1 × ST12, a total of 1418 SNPs were obtained. However, in the cross combination of WAB56-50 × ST6, only a total of 828 SNPs were obtained. This could be due to the genetic similarity between WAB56-50 and ST6. These two lines share a *tropical japonica* genetic background. In terms of RPGR analysis, variation between and within the genetic backgrounds were observed. Theoretically, the RPGR of BC_3_ generation should be at 93.75%. The best performing lines in our study showed an RPGR that is within and higher than the theoretical RPGR. However, we observed some lines that had an RPGR lower than the theoretical. For example, NERICA4 × ST6 lines were observed to have the lowest maximum RPGR at 87.7%. The low RPGR observed in these lines could have been avoided if the analysis was carried out as early as BC_1_ generation [19,41]. Nowadays, background selection at an early generation through GBS is cost-efficient for MAB programs.

The results of our study showed phenotypic variation on other agronomic traits. For example, some lines carrying the *Gn1a*, *WFP*, and combination of both alleles showed a significant improvement in SBPP, PF, and TGW (Supplementary Tables S1–S6). In addition, lines carrying *Gn1a*, *WFP*, and combination of both alleles showed a significant increase in PH and decrease in PL and TNPP. These phenomena could be due to non-targeted introgression or interaction of QTLs with other regulatory genes. In addition, the phenotypic variations that were observed in the aforementioned traits varied significantly across seasons. These variations could be attributed to the genetic background of the materials used in the study. The environmental changes, especially day length and temperature, during the wet and dry season may have also contributed to these seasonal variations. A careful evaluation of the succeeding trials is necessary to confirm these phenotypic and seasonal variations.

For farmers to adopt these new lines, agronomic traits must be comparable with the recurrent parents. The selected WISH lines developed in this study are by far comparable with their respective recurrent parents. Even though the *WFP* lines have fewer TNPP, the general agronomic morphology still coincides with the concept of new plant type [42] (low tillering capacity; 200–250 grains per panicle 110–130 days growth duration). In addition, although yield per plant was not evaluated in this study, it would be necessary to determine the performance of these lines in the actual farmer’s field. Additionally, further improvement in field agricultural practices is necessary to fully draw the genetic potential of the WISH lines.

The utilization of yield and yield-related QTLs has been a challenge for most breeding programs due to its complexity brought by genotypic and environmental effects. In this study, we demonstrated the success of utilizing MABB to introgress and stack the *Gn1a* and *WFP* alleles. In addition, the current study used GBS technology to conduct background genome analysis of the developed lines. To the best of our knowledge, this is one of the first attempts to investigate the effects of *Gn1a* and *WFP* in systematically produced introgression lines. However, the evaluation of WISH lines in various environments, especially in SSA, is needed to assess its stability.

## 4. Materials and Methods

### 4.1. Plant Materials

**Figure 9:**
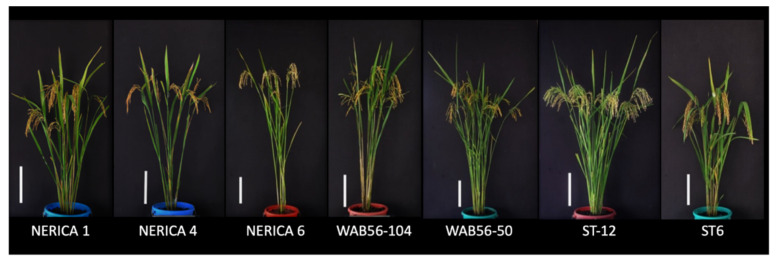
Gross morphology of NERICA and WAB rice cultivars, and the *Gn1a* and *WFP* rice line donors ST12 and ST6. Scale bar = 20 cm.

The New Rice for Africa (NERICA) cultivars (NERICA1, NERICA4, and NERICA6) and progenitor lines (WAB56-50 and WAB56-104) were used as the recurrent parents in this study (Figure 9). The donor rice lines ST12, a high-yielding rice line in the background of *indica* [15]**,** and ST6, a high-yielding rice in the background of *japonica* [43]**,** were used as the donor of *Gn1a* and *WFP.* The donor germplasms were obtained from the stocked rice collections of the Togo field at Nagoya University, Japan.

### 4.2. Development of WISH Lines by MAS

**Figure 10:**
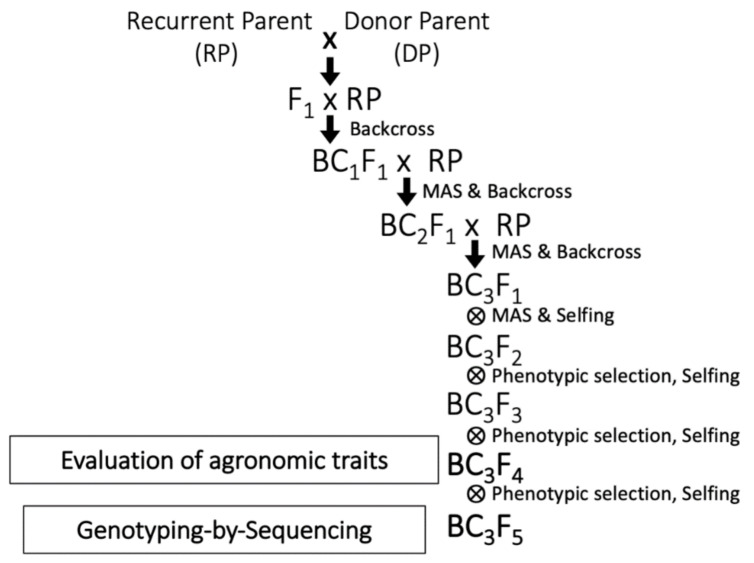
Marker-assisted backcross breeding scheme of *Gn1a* and *WFP* introgression in NERICA and WAB rice cultivars.

The initial breeding lines were generated at Nagoya University, Japan in 2012. In 2013, the backcross population at BC_1_F_1_ generation were exported to IRRI for further backcrossing and generation advancement. The lines were backcrossed for three consecutive seasons (DS 2014, WS 2014, and DS 2015), generating BC_3_F_1_ lines. MABB was carried out at BC_2_F_1_ and BC_3_F_1_ to assure that the donor alleles of *Gn1a* and *WFP* were present in each generation using the DNA markers RM3360 for *Gn1a*, and RM3452 and RM5493 for *WFP* [31]. All BC_3_F_2_ lines were screened to obtain lines that were homozygous for the donor alleles in the target loci. Selected lines were advanced to BC_3_F_4_ and BC_3_F_5_. At BC_3_F_4_ and BC_3_F_5_, lines were evaluated phenotypically and genotyped by GBS (Figure 10). All lines developed in this research are part of the WISH project. These lines are referred to as WISH lines.

### 4.3. Foreground Selection for Gn1a and WFP Alleles

The RM markers used to identify lines carrying *Gn1a* and *WFP* alleles are summarized in Table 2. Genomic DNA from each sample was extracted using the TPS method [44]. PCR analysis was performed using Veriti^TM^ 384-well thermal cycler (Thermo Fisher Scientific, Waltham, MA, United States) with an initial denaturation of 95 °C for 5 min; 35 cycles at 95 °C for 30 s; annealing at 55 °C for 30 s; and extension at 72 °C for 30 s with a final extension at 72 °C for 7 min. The PCR product was separated on 3% agarose gel at 200 v for 40 min. Gel results were visualized by using a UVIdoc-HD2/20M (Cleaver Scientific Ltd., Warwickshire, United Kingdom) Gel Documentation System.

### 4.4. Genotyping-by-Sequencing (GBS)

To determine the recurrent genome recovery, the population was subjected to GBS, as described by Poland et al. [33]. In summary, genomic DNA (200 ng) of individual lines were double-digested with *Kpn*I–*Msp*I (New England Biolabs Inc., Ipswich, MA, USA) and ligated with unique barcoded adapters. Individual barcoded samples were pooled into a single tube. The sequencing of the library was carried using Illumina HiSeq X Ten (Illumina, Inc., San Diego, CA, USA).

TASSEL-GBS 5.0 [45] was used for informatics to process the GBS data. Reads were aligned to the IRGSP V1.0 *O. sativa* Nipponbare reference genome using BWA [46,47]. The parameters for the SNP caller plugin were minor allele frequency (MAF) of 0.02 and minimum locus coverage (mnLCov) of 0.1. The reads that were obtained after the initial processing were further filtered using the VCF tools [48] and awk scripts written by the authors. In summary, filtering was based on polymorphism between parental alleles, a minimum depth of 6, and a maximum missing value of 30%.

### 4.5. Recurrent Parent Genome Recovery (RPGR) Analysis

The RPGR of the lines was computed using Graphical Genotyper (GGT 2.0) software, and graphical genotypes were created using Microsoft Excel [49,50]. Only polymorphic markers between the donor and recurrent parents were included in the analysis. The homozygous recurrent parent allele, donor parent allele, and heterozygous allele were denoted as A, B, and H, respectively.

### 4.6. Field Trials and Agronomic Evaluation

Field experiments were conducted at the hybridization block (HB) (14°10′18.4″ N 121°15′33.3″ E) of IRRI. The WISH lines were planted with twelve plants per line and 20 cm × 20 cm plot spacing. The field management was based on the standard practice at the research institute. To summarize, fertilizer was applied thrice; basal dressing at final harrowing; first topdressing 21–25 days after seeding; second topdressing 30–35 days after seeding. Pests and diseases were controlled using chemicals to avoid yield loss in plants.

At maturity, five plants that were homozygous at the target loci were selected in each line and evaluated for their agronomic traits. The HD was determined from seeding day until 50% of plants per line were flowering. PH was measured from the base of the main stem (above ground) to the tip of the primary panicle. TNPP was recorded as productive tillers in a single plant. PL was measured from the base of the peduncle to the tip of the primary panicle. The PBPP is the total number of branches directly from the peduncle. The SBPP is the number of branches from the PBPP. The NSPP in this study was measured as the total number of spikelets present in each panicle. The PF was measured as the total number of filled grains/total number of grains per panicle × 100. The TGW was measured as the total weight of 1000 grains.

### 4.7. Data Analysis

The recorded agronomic data were analyzed using Statistical Tool for Agricultural Research software (STAR) v2.01 [51]. To determine the significant differences between recurrent parent and WISH lines, one-way analysis of variance (ANOVA) and Tukey’s test at 95% confidence level (*p* < 0.05) were used.

## Figures and Tables

**Table 1:** Total number of SNPs and recurrent parent genome recovery range of WISH lines**.**

Genetic Background	Total Number of SNPs	Recurrent Parent Genome Recovery Range of BC_3_F_4_ Population (%)
NERICA1 × ST12	1418	85.4–98
NERICA4 × ST6	1175	84.5–87.7
NERICA6 × ST12	1658	94.8
WAB56-104 × ST6	1614	77.7–96.9
WAB56-104 × ST12	1064	80.6–93.1
WAB56-50 × ST12	1873	88.3–96.7
WAB56-50 × ST6	828	81.2–95.3

**Table 2:** List of microsatellite markers used to monitor the introgression of *Gn1a* and *WFP* in recipient lines.

Marker	Forward Primer Sequence	Reverse Primer Sequence	Target Gene
RM3360	ACTTACACAAGGCCGGGAAAGG	TGGTAGTGGTAACTCTACTCCGATGG	*Gn1a*
RM3452	TGGACTTGGTCTCTCCAAACTCC	CAGTATGTGTTGGTGGGTCAAGC	*WFP*
RM5493	GCGGTAACAAACCAACCAACC	AAAGCAGGACACAGTCACACAGG	*WFP*

## Supplementary Materials

The following are available online at https://www.mdpi.com/article/10.3390/plants10050844/s1, Table S1: Mean ± SD values of the agronomic traits of WISH lines with Gn1a-ST12 introgression title, Table S2: Mean ± SD values of the agronomic traits of WISH lines with Gn1a-ST6 introgression, Table S3: Mean ± SD values of the agronomic traits of WISH lines with WFP-ST12 introgression, Table S4: Mean ± SD values of the agronomic traits of WISH lines with WFP-ST6 introgression, Table S5: Mean ± SD values of the agronomic traits of WISH lines with Gn1a+WFP-ST12 introgression, Table S6: Mean ± SD values of the agronomic traits of WISH lines with Gn1a+WFP-ST6 introgression.
